# Antidiabetic effects of *Eryngium billardieri* hydrosol in the treatment of type 2 diabetic patients: A double-blind randomized clinical trial

**DOI:** 10.22038/AJP.2022.21175

**Published:** 2023

**Authors:** Zahra Hamami Chamgordani, Mohammad Mazaheri, Bijan Iraj, Hojjat Baghshahi, Fakhri Sabouhi

**Affiliations:** 1 *Department of Adult Health Nursing, Faculty of Nursing and Midwifery, Isfahan University of Medical Sciences, Isfahan, Iran*; 2 *Persian Medicine, Faculty of Medicine, Isfahan University of Medical Sciences, Isfahan, Iran*; 3 *Isfahan Endocrine and Metabolism Research Center, Isfahan University of Medical Sciences, Isfahan, Iran*; 4 *Barij Medicinal Plants Research Center, Kashan, Iran*; 5 *Nursing and Midwifery Care Research Center, Isfahan University of Medical Sciences, Isfahan, Iran*

**Keywords:** Antidiabetic activity, Blood lipid profile, Glycemic index, Glycosylated hemoglobin, Medicinal plant

## Abstract

**Objective:**

Medicinal plants with fewer side effects and low cost than synthetic medicines are increasingly advised to treat diseases. The present study aimed to identify *Eryngium billardieri* compounds and evaluate the plant’s effects on hyperglycemic and hyperlipidemia indices, and liver, and kidney function.

**Materials and Methods:**

Following identification of *Eryngium billardieri* using GC/MS method, 72 participants were randomly divided into two groups (n=36 per group), receiving oral hypoglycemic medication (metformin) with or without 50 ml hydrosol twice a day for three months as intervention and placebo control, respectively. Body mass index (BMI), systolic and diastolic blood pressure, fasting blood sugar (FBS), glycosylated hemoglobin (HbA1c), total cholesterol, triglyceride, HDL-C, and LDL-C levels were measured at the beginning and end of the experiment. Also, aspartate transaminase, alanine transaminase, blood urea nitrogen, and creatinine levels were measured to assess adverse effects on liver and kidney functions.

**Results:**

The main components were terpenes with 46.69% of the total ingredients of *E. billardieri* essential oil. Other prominent compounds identified included octanoic acid (12.14 %) and isoxazole (6.72 %). Intergroup changes in blood parameters showed that *E. billardieri* hydrosol for three months could significantly reduce HbA1C and blood cholesterol levels but did not affect other measured parameters. Also, there were no adverse effects on kidney or liver function.

**Conclusion:**

The present findings showed that the consumption of 50 ml of *E. billardieri* hydrosol as a complementary treatment in diabetic patients reduced HbA1C and cholesterol levels without adverse effects on the liver or kidneys functions.

## Introduction

Diabetes mellitus (DM) is a common chronic metabolic disease associated with a significant risk of death (Zheng et al., 2018 ▶). Diabetes affected 463 million people worldwide in 2019, a number predicted to increase to 578 million by 2030 and 700 million by 2045 (Saeedi et al., 2019 ▶). Diabetes mellitus type 2 (DMT2) accounts for more than 90% of diabetics (Attele et al., 2002 ▶). DMT2, known as adult-onset diabetes or non-insulin-dependent diabetes, is distinguished by a relative lack of insulin due to dysfunction of pancreatic cells or insulin resistance in target organs (Chatterjee et al., 2017 ▶). This impairment leads to persistent hyperglycemia which leads to numerous consequences such as cardiovascular disorder, neuropathy, blindness, renal disease, and peripheral gangrene (Chang et al., 2013 ▶). On the other hand, the development of such health complications reduces the quality of life of DMT2 patients (Wexler et al., 2006 ▶).

Despite significant medical development, the treatment of DMT2 and the reduction of its side effects are still a serious health problem (Wadkar et al., 2008 ▶). Due to the chemical composition and side effects of many synthetic drugs, the willingness to take conventional medicines is decreased, and patients tend to use complementary therapies (Ranasinghe et al., 2012 ▶). As a result, the usage of herbal treatments in both developing and developed countries is increasing (WHO, 2019 ▶; Alqathama et al., 2020 ▶).

*Eryngium billardieri *(*E. billardieri*) belongs to the Apiales order and in the Apiaceae (Umbelliferae) family, and it is widely utilized as a medicinal plant for the treatment of inflammatory diseases all over the world (Sefidkon et al., 2004 ▶; Landoulsi et al., 2016 ▶). The most important components of *E. billardieri * are alkaloids, flavonoids, saponins, kaempferol, terpenoids, coumarone, chlorogenic acid, caffeic acid, and β-carotene (Kosiński et al., 2019 ▶). Anti-diabetic, anti-inflammatory, antioxidant, cytotoxic, anti-apoptotic, anti-bacterial, anti-fungal, and anti-malarial effects are among the biological effects associated with these substances (Yesilada et al., 1989 ▶; Sefidkon et al., 2004 ▶; Küpeli et al., 2006 ▶; Tofighi et al., 2014 ▶; Erdem et al., 2015 ▶). The root and aerial parts of this plant are utilized in Iranian traditional medicine for various diseases such as diabetes, inflammatory disorders, rheumatism, sinusitis, urinary infections, scorpion bites, and goiter and for wound healing (Sefidkon et al., 2004 ▶; Esmaeili et al., 2016 ▶; Khani et al., 2021 ▶).

It has been reported that *E. billardieri *extract improved glucose uptake and metabolic parameters in streptozotocin/nicotinamide-induced diabetic rats (Khani et al., 2021 ▶). Also, *E. billardieri *has been considered a potential treatment for pancreatic cancer due to its apoptotic and cytotoxic properties (Roshanravan et al., 2018 ▶). However, the effects of long-term consumption of *E. billardieri *on hyperglycemic and hyperlipidemic indices, as well as its possible side effects in humans are unknown. Therefore, this study aimed to identify *E. billardieri* essential oil compounds and evaluate the effects of *E. billardieri* hydrosol on DMT2 treatment as a supplement.

## Materials and Methods

**Plant material and extraction of essential oils**

The *E. billardieri *hydrosol was purchased from a local market in Isfahan, Isfahan province, Iran. A voucher specimen has been deposited in the Herbarium of the Agriculture Department of Barij Essence Pharmaceutical Company under number 243-1. The essential oil of *E. billardieri* hydrosol was extracted as described by Moravej et al. (Moravej et al., 2016 ▶) . The essential oil of the sample was extracted using a liquid extractor in three steps. Initially, 500 g of the sample was extracted three times in a decanter funnel three times each time with 50 ml of n-pentane for 20 min at room temperature. The pentane phases were then separated and mixed. The final pentane phase was dehydrated with 2 g of sodium sulfate and placed in an oven in a dry beaker at 40°C. After solvent evaporation, the final sample was collected for gas chromatography (GC) and gas chromatography-mass spectrometry (GC/MS) analysis.

**Gas chromatography-mass spectrometry**

The Agilent 6890 Gas Chromatograph was used to conduct chromatography gas mass spectrometry (GC/MS) analysis (Agilent Technologies model 5973N mass detector, Santa Clara, CA, USA) (Massada, 1976 ▶). The GC was embedded with an HP-5ms capillary column [phenylmethylsiloxane, length 60 m × inner diameter 0.25 mm, Agilent technologies (60-325/350°C)]. The oven temperature was raised from 50°C to 250°C with an increase of 3°C per minute and held for 10 minutes. Helium was chosen as the carrier gas, and the flow rate was adjusted at a rate of 1 ml per minute. The mass spectrometer operated in electron impact ionization (EI) mode at 70 eV. The mass spectrum of essential oil components was compared to the Wiley Library published ones (Kikowska et al., 2020 ▶), and NIST Mass Spectral Database from https://webbook.nist.gov/chemistry/cas-ser.

**Study design**

This study was conducted as a double-blind randomized block clinical trial on type 2 diabetics at Isfahan Endocrine and Metabolism Research Center, Sedigheh Tahereh Medical Research Complex, Isfahan, Iran. The procedure was validated by the Medical Ethics Committee of Isfahan University (approval code: IR.MUI.RESEARCH.REC.1398.506). Furthermore, the trial was registered by the Iranian Registry of Clinical Trials with the following code: IRCT20150620022821N2. A total of 67 patients with an age range of 30-60 with criteria such as DMT2 and use of oral hypoglycemic drugs for at least three months, no insulin injection, at least two years of diabetes, body mass index less than 30, no restrictions on the use of medicinal plants, interested in participating in the study, not using other methods of traditional medicine, were selected by a physician. This study excluded pregnant or lactating women or those with glycosylated hemoglobin (HbA1c) levels in the range of 6.5 to 5.5% in the last three months. Exclusion criteria included hospitalization, intolerance to therapy (expression of discomfort or sensitivity to the drug), pregnancy, refusal to continue taking the herbal supplement or switching from an oral to an injectable pharmaceutical regimen. All participants were asked not to change their current herbal supplement or diet during the study. The medicine or placebo was prescribed by a trained physician twice a day, each time at a dose of 25 ml before breakfast and lunch for three months. Metformin was administered to both groups simultaneously, according to a physician consult. The placebo was supplied in similar packaging to the herbal supplement and contained 10% hydrosol of *E. billardieri *to mimic the same flavor. Patients were contacted once a week to check on their health. Body mass index (BMI), systolic and diastolic blood pressure, fasting blood sugar (FBS), HbA1c, total cholesterol, triglyceride, HDL-C, and LDL-C levels were all measured at the beginning and end of the study. The aspartate transaminase, alanine transaminase, blood urea nitrogen, and creatinine levels were measured to assess any adverse effects. All biochemical analyses were performed by the Central Laboratory. HbA1c levels were determined by HPLC (Tosoh HLC-723 analyzer). All other blood sample parameters were analyzed using Audit Diagnostics Kits (sensitivity=1 mg/dl, Ireland) using the Hitachi 917 automated analyzer.

The patients’ weight was determined using a digital scale Seca with an accuracy of 100 g in the morning on an empty stomach and with minimum clothing. The height was measured using a fixed-to-the-wall tape meter in a standing position without shoes, heels attached to the wall, and a straight head. Afterward, BMI was calculated in kilograms per square meter.

**Sample size**

The sample size was calculated using the following formula (Yalcin et al., 2008 ▶): 

$N=\frac{{\left(Z_{1-\frac{\alpha }{2}}+Z_{1-\beta }\right)}^{2}({S_{1}}^{2}+{S_{2}}^{2})}{d^{2}}$

S_1_= 23.2, S_2_= 26.2, d= 20

According to a previous study, we needed 64 participants in each group (*E. billardieri* and placebo groups) to complete the trial based on a significant mean difference of fasting plasma glucose (Khani et al., 2021 ▶), with 95% confidence level, 90% power, and a probable drop-out rate of 10%. 

**Size of randomized blocks**

After explaining the experiment, patients completed an informed consent form and were allocated into *E. billardieri* hydrosol or placebo groups by block randomization, using a permuted block method (with a block size of four) concealed in sequentially numbered envelopes. A trained nurse blindly allocated patients to the groups. The procedure was continued until all of the 72 eligible patients were assigned to the blocks. After randomization, 36 patients were assigned to the *E. billardieri* group and 36 patients were assigned to the placebo group. 

**Figure 1:**
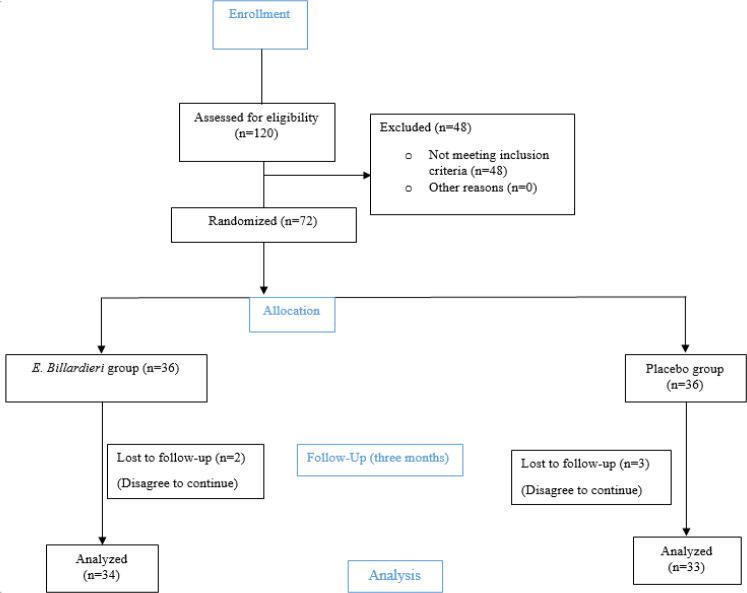
The CONSORT flow diagram of participants

During the follow-up period, two and three patients were respectively excluded from the intervention and placebo groups due to refusal to continue the study. Finally, 34 and 33 patients remained in the intervention and placebo groups, respectively (Figure 1).

**Study outcomes**

The primary outcome of this study was baseline and end-of-trial changes in HbA1c, FBS, and total cholesterol values from baseline to the end of the trial. Secondary outcomes included BMI, triglyceride (mg/dl), systolic and diastolic blood pressure, HDL, LDL, liver profile (AST, ALT), and renal profile (serum creatinine).

**Statistical analysis**

The normal distribution of data was checked by the Kolmogorov-Smirnov test. The data were analyzed using SPSS v17.0 software (SPSS, Inc.). A paired t-test was used to compare before and after the intervention. Also, an independent t-test was performed to examine the differences between the two groups. p<0.05 values were considered statistically significant.

## Results

***E. billardieri*** **essential oil components**

**Figure 2:**
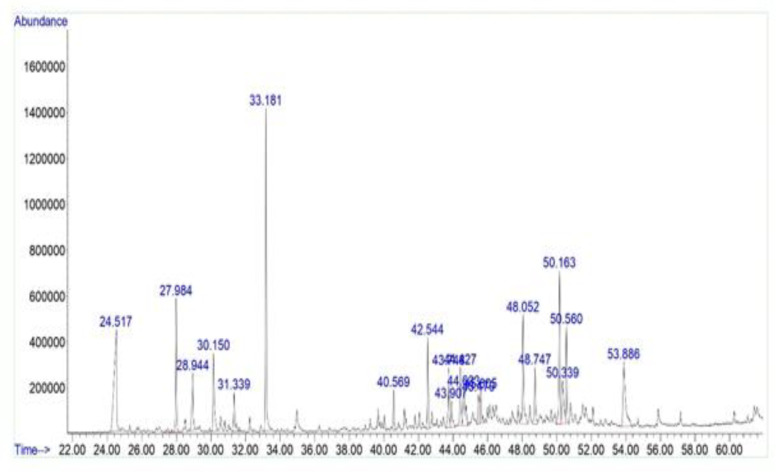
Typical GC-MS total ion current (TIC) chromatograms of *Eryngium billardieri*

GC/MS analysis of *E. billardieri *essential oil showed that terpenes constituted the major part (46.69%) of the total ingredients, including monoterpenes (carvone 6.11% and thymol 4.91%), terpenoids (dihydroactinidiolide 2.15%), and sesquiterpenes (spathulenol 4.02%, α-elemene 3.35%, longifolene 3.86%, caryophyllene 2.07%, gurjunene 2.03%, epiglobulol 6.72%, longifolenaldehyde 3.75%, and clovanediol 7.72%). Other prominent compounds identified included octanoic acid (12.14 %) and isoxazole (6.72 %) (Figure 2 and Table 1).

**Demographic characteristics**

Three months following the start of the trial, the data was examined and compared to the baseline data. Table 2 shows the demographic characteristics of both groups at the start of the trial. There was no statistically significant difference between the two groups in these characteristics.

**The blood parameters and body mass index**

The blood parameters and BMI before and after the intervention, and inter-group changes are shown in Table 3. BMI decreased significantly in the intervention group (p*<*0.05) at the end of the intervention, but there was no significant difference in the placebo group; there was also no significant difference in the mean changes in BMI between the two groups. The placebo group had no difference in mean systolic and diastolic blood pressure between before and after the trial, whereas the intervention group had a substantial difference (p*<*0.05). However, there were no differences in systolic and diastolic blood pressure variations between the two groups. FBS levels in the intervention and placebo groups were significantly lower at the end of the trial compared to the beginning (from 216.16±79.92 to 165.79±53.07 and 159.75±21.03 to 132.50±40.43 mg/dl, respectively) (p*<*0.05). As a result, there was no significant difference in changes in FBS between the two groups. The mean HbA1c level did not change significantly during the trial in the placebo group but decreased dramatically in the intervention group after the intervention (p<0.05). These changes in HbA1c levels between the two groups were significant (p*<*0.05). Total cholesterol level showed a similar pattern of HbA1c. The difference in mean triglyceride level in the placebo group was not significant after the intervention, although there was a borderline significant difference in the intervention group (p=0.05); nevertheless, the changes in mean triglyceride level between the two groups did not indicate a significant difference. After the experiment, there was no significant difference in mean HDL-C level between the intervention and placebo groups, nor were there any changes in mean HDL-C level between the two groups. After the intervention, the mean LDL-C level in the intervention group reduced at p=0.05. However, there was no significant difference between the intervention and placebo groups. After three months, the AST and ALT levels did not differ between the two groups. Also, there was no significant difference in the mean or changes in renal biochemical markers (BUN and creatinine).

**Table 1:** The components of *Eryngium billardieri *essential oil

**No.**	**Components**	**RT**	**Content (%)**
1	Octanoic acid	24.517	12.14
2	Carvone	27.984	6.11
3	Nonanoic acid	28.944	3.53
4	Thymol	30.150	4.91
5	2,4,6-Trimethylbenzaldehyde	31.339	2.66
6	2,4,5-Trimethylbenzaldehyde	33.181	15.00
7	Dihydroactinidiolide	40.569	2.15
8	Spathulenol	42.544	4.02
9	Butylphosphonic acid	43.746	3.16
10	1,3-benzodioxole	43.907	1.74
11	α-Elemene	44.427	3.35
12	Longifolene	44.633	3.86
13	Caryophyllene	45.479	2.07
14	Gurjunene	45.605	2.03
15	Epiglobulol	48.052	6.72
16	Annulene	48.747	3.25
17	Isoxazole	50.163	7.69
18	Longifolenaldehyde	50.339	3.75
19	2-Cyclohexen-1-one	50.560	3.97
20	Clovanediol	53.886	7.72
Overall percentage			99.83
Trace compounds			0.17

RT, identification based on retention time

**Table 2:** The demographic characteristics of patients in placebo and *Eryngium billardieri *treated groups (mean±SD)

Parameter	*E. billardieri*	Placebo	p-value
Age (years)	49.74±5.91	50.17±8.94	0.87
Sex (male/ female)	25 F/9 M	20 F/13 M	0.49
BMI	26.66±1.24	26.92±1.08	0.57
Average number of pills taken per day	3.10±0.56	2.94±0.80	0.15

**Table 3:** The blood parameter levels and body mass index in the Placebo and *E. billardieri *treated groups (mean±SD)

Parameter	*E. billardieri* (n=34)			Placebo (n=33)			p-value Difference of difference ^b^
	Beginning	After 3 months	p-value	Beginning	After 3 months	p-value^ a^	
BMI (kg/m^2^)	26.66±1.25	26.38±1.04	0.04	26.92 ±1.08	26.86±1.18	0.74	0.26
Systolic blood pressure (mm Hg)	12.21±1.31	11.68±0.94	0.04	12.17±1.53	11.67±0.98	0.14	0.95
Diastolic blood pressure (mm Hg)	7.84± 0.50	7.68±0.48	0.02	7.75±0.62	7.67±0.49	0.72	0.77
FBS (mg/dl)	216.16±79.92	165.79±53.07	0.0001	159.75±21.03	132.50±40.43	0.008	0.14
HbA1c (%)	9.90± 2.14	8.86±2.01	0.0001	8.12±1.29	7.9±1.46	0.28	0.001
Total cholesterol (mg/dl)	188.32±45.90	167.10±42.69	0.03	160.92±40.78	165.50±33.22	0.55	0.05
Triglyceride (mg/dl)	191.26±91.30	167.63±65.38	0.05	115.75±24.22	112.50±24.90	0.56	0.19
HDL-C (mg/dl)	43.89±10.04	46.15±9.43	0.07	47.33±13.67	51.67±16.40	0.14	0.43
LDL-C (mg/dl)	112.38±41.90	99.05±39.42	0.05	99.83±40.03	98.58±39.27	0.82	0.19
AST (U/L)	22.84±5.26	23.32±5.19	0.48	21.00±3.69	23.00±4.55	0.18	0.28
ALT (U/L)	21.16±5.64	21.63±5.15	0.43	21.00±4.13	21.42±3.34	0.36	0.94
BUN (mg/dl)	12.47±2.12	12.51±2.27	0.90	14.42±3.36	13.92±2.31	0.64	0.63
Creatinine (mg/dl)	0.85±0.11	0.86±0.15	0.51	0.82±0.13	0.85±0.11	0.22	0.70

^a^ p-Value reported based on Paired- samples T test. ^b^ p-Value reported based on Independent-Samples T test

## Discussion

In patients with DMT2, the effect of *E. billardieri *hydrosol on glycemic and lipid indices, as well as its side effects, is yet unclear. This study was carried out to evaluate the anti-diabetic effect of *E. billardieri *hydrosol on patients with DMT2. 

According to investigations done on several species of the *Eryngium* genus, their similar compounds to our study include thymol, pinocarvone, spathulenol, trimethylbenzaldehyde, elemene, caryophyllene, gurjunene, and globulol. However, the quantity of chemicals found in our study differs from theirs, and this variation can be attributed to the varied cultivated geographical regions and species (Sefidkon et al., 2004 ▶; Kremer et al., 2021 ▶). 

Our research showed that terpenes are a significant constituent of *E. billardieri *essential oil. Terpenes have been found to provide appropriate protection to the body through direct suppression of reactive oxygen species and regulation of the endogenous antioxidant system under oxidative stress conditions associated with the pathological progression of diabetes (Gonzalez-Burgos and Gómez-Serranillos, 2012 ▶). Terpenoids reduce circulating lipid levels and increase insulin sensitivity by modulating glucose transporter type-4 translocation and dual activation of PPAR-alpha and gamma (Tenenbaum et al., 2005 ▶; Huang and Czech, 2007 ▶; Goto et al., 2010 ▶; Kuo et al., 2016 ▶). Our findings showed that thymol and carvone were major terpene compounds identified in the essential oil of *E. billardieri*. It has been demonstrated that thymol decreases plasma glucose and HbA1c, improving insulin resistance (Tenenbaum et al., 2005 ▶; Saravanan and Pari, 2015 ▶). Carvone has been demonstrated to improve insulin secretion, reverse abnormalities in glycoprotein levels, and enhance key carbohydrate metabolism enzymes in the liver tissue in diabetic rats (Muruganathan et al., 2013 ▶; Muruganathan and Srinivasan, 2016 ▶; Abbas et al., 2020 ▶). Other main compounds in *E. billardieri *essential oil are octanoic acid and isoxazole. In humans and rats, octanoic acid has been confirmed to increase glucose oxidation and glucokinase expression, potentiating glucose-stimulated insulin production (Zhang et al., 2019 ▶). On the other hand, octanoic acid promotes positive feedback by the olfactory receptor 15 during glucose-stimulated insulin production (Leem et al., 2018 ▶). Isoxazole derivatives have recently been shown to reduce insulin resistance in HepG2 cells by boosting glucose absorption, and that is why they are classified as anti-diabetic medicines (Sysak and Obmińska-Mrukowicz, 2017 ▶; Nie et al., 2020 ▶).

Research has also shown that natural antioxidants reduce oxidative stress in pancreatic tissue, which slows the progression of diabetes. Antioxidants prevent damage to various cell components by neutralizing reactive oxygen compounds. They can eventually enhance the activity of insulin receptors and hepatocyte antioxidant enzymes, minimize lipid peroxidation, and stimulate glucose transport (Ghorbani, 2017 ▶).

In this study, FBS decreased in both placebo and *E. billardieri* groups during the three months. It could be due to the change in nutritional status or physical activity in the control group. Studies have shown a significant relationship between levels of HbA1C and FBS. It is shown that HbA1C is more sensitive than FBS to monitor the progression of diabetes and was superior to FBS when used as a biomarker to diagnose diabetes (Chung et al., 2017 ▶).

Our results revealed that *E. billardieri *hydrosol significantly reduced HbA1C and cholesterol levels compared with the control group in patients with DMT2 after three months. The presence of specific compounds such as flavonoids and tannins in this plant may be linked to its hypoglycemic and hypolipidemic effects (Oyedemi et al., 2012 ▶). Rabiee et al. reported that short-term consumption of *E. billardieri *hydrosol reduced plasma glucose levels in diabetic men, although it did not affect cholesterol (Rabiee et al., 2017 ▶). This difference can be due to the duration of the use of hydrosol. However, they showed that insulin production from pancreatic beta cells was increased by short-term *E. billardieri* supplementation. This supplement can affect pancreatic beta cells and increase insulin production. On the other hand, it can increase the number of insulin sensitivity by increasing the number of GLUT4 receptors in the cell membrane and increasing the affinity of these receptors for insulin. Their results are in line with the results of reducing the HbA1C levels with the consumption of *E. billardieri* hydrosol observed in the present study.

Rats fed with *E. billardieri *extract had considerably lower cholesterol, triglycerides, and LDL levels (Zarei et al., 2015 ▶). Another investigation found that feeding *E. billardieri *to male rats decreased glucose levels, lipid profiles, and liver enzymes but raised HDL levels to normal levels (Khani et al., 2021 ▶). The presence of saponins and alkaloids in the hydrosol of *E. billardieri *regulates the fat profile by inhibiting intestinal cholesterol absorption and boosting cholesterol secretion through biliary excretion, resulting in a reduction in the level of plasma cholesterol (Eguchi et al., 2013; ▶ Banda et al., 2018 ▶). 

BUN and creatinine are critical indicators for metabolic disorders and patient nutritional status. BUN and creatinine levels have an inverse relationship with renal function (Griffin et al., 2019 ▶). There was no difference in BUN or creatinine levels between the placebo and treatment groups, indicating that *E. billardieri *hydrosol had no adverse effect on renal function. ALT and AST are two critical enzymes in amino acid/glucose metabolism pathways that play a crucial role in amino acid metabolism in muscles and gluconeogenesis in different tissues. Functionally, AST and ALT are essential biochemical linkages between carbohydrate and protein metabolism. Elevated serum levels of ALT and AST reflect liver dysfunction or injury (Kunutsor et al., 2014 ▶).

Because there was no change in AST or ALT levels in the current investigation, it can be stated that *E. billardieri *hydrosol does not affect liver function in DMT2 patients. Laboratory studies support the findings of the present study, which show that *E. billardieri *has no detrimental effect on kidney or liver function (Zarei et al., 2015 ▶; Khani et al., 2021 ▶).

The current study found that* E. billardieri *essential oil has known anti-diabetic compounds. Daily consumption of 50 ml of *E. billardieri *hydrosol can decrease HbA1C and blood cholesterol as a complementary treatment in type 2 diabetic patients. Clinical studies are suggested to evaluate the benefits and limitations of higher doses of *E. billardieri* hydrosol over a quarterly period. 

Patients refused to attend the therapy clinic every day due to the coincidence of the present trial with the prevalence of Covid-19, thus FBS levels like other parameters were assessed at the beginning and end of the research.

## Conflicts of interest

The authors have declared that there is no conflict of interest.
